# Research on alkali metal-modified Pd catalyst for oxygen removal from propylene

**DOI:** 10.3389/fchem.2022.987556

**Published:** 2022-09-16

**Authors:** Jinchong Zhao, Jie Jiang, Song Wen, Jing Zhang, Changsheng Zhang, Nan Sheng, Wei Liang, Bing Sun, Wei Xu, Zhe Yang, Yuan Pan

**Affiliations:** ^1^ State Key Laboratory of Safety and Control for Chemicals, SINOPEC Research Institute of Safety Engineering Co., Ltd., Qingdao, China; ^2^ State Key Laboratory of Heavy Oil Processing, China University of Petroleum (East China), Qingdao, China

**Keywords:** Pd catalyst, alkali metal modification, β-PDH, oxygen removal, propylene purification

## Abstract

A series of alkali metal (Li, Na, and K)-modified Pd catalysts and Pd/Al_2_O_3_ were prepared and used to remove oxygen in a propylene flow with hydrogen’s existence. The results showed that the alkali metals could enhance the performance of the Pd catalysts and the effect followed the order of K > Na > Li. X-Ray diffraction (XRD), N_2_-physisorption, transmission electron microscopy (TEM), hydrogen temperature programmed reduction (H_2_-TPR), and X-ray photoelectron spectroscopy (XPS) were carried out to investigate the alkali metal-modified Pd catalysts and the promotional effect mechanism was explained. The results showed that alkali metal modification increased the electron density of Pd atoms to induce the negatively charged Pd species, which could enhance the adsorption of oxygen while weakening the adsorption of propylene, and then enhance the performance of the modified catalysts for oxygen removal from unsaturated hydrocarbon. The Pd-K/A catalyst performed the best on both oxygen removal and propylene hydrogenation inhibition.

## Graphical Abstract

To recycle the propylene safely from a gas mixture consisting of propylene and oxygen, alkali metal-modified Pd catalysts were used to remove oxygen from the gas with the help of hydrogen.
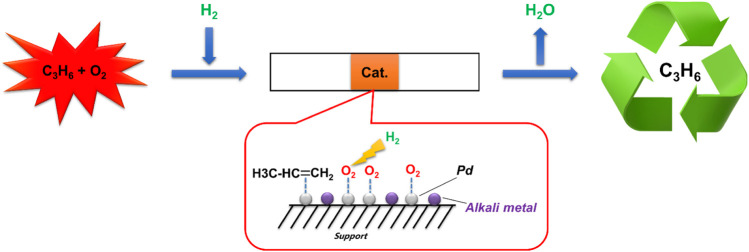

## Introduction

Propylene oxide (PO) is an important petrochemical product with a great market in the chemical industry. Hydrogen-peroxide-to-propylene-oxide technology (HPPO) for the production of propylene oxide has great advantages in environmental protection, which has attracted great attention in recent years (Climent et al., 2011; Lin et al., 2016; Li et al., 2022). The tail gas of the HPPO process contains large amount of oxygen produced by hydrogen peroxide decomposition, which makes the tail gas too dangerous to be separated and recycled (Movileanu et al., 2012; Razus et al., 2009; Razus et al., 2007). Some methods have been reported for solving this problem by removing the oxygen via catalytic reactions. Joaquim Henrique Teles et al. reported a catalyst consisting of LiCl and TiO_2_, which could catalyze the oxidative dehydrogenation reaction between propane and oxygen in the HPPO exhaust at 585°C to remove oxygen (Teles et al., 2004). Lv et al. reported a Mn-contained adsorbent for oxygen removal, ethylene or propylene with little oxygen could be purified at 200°C by the adsorbent (Lv et al., 2005). Schindler et al. developed a Pt-Sn/α-Al_2_O_3_ catalyst, by whose effect oxygen and hydrogen could react to produce water at a temperature between 250–300°C, while a small amount of propylene might react with hydrogen (Schindler et al., 2011). Although there have been many technologies for oxygen removal in gas mixtures, most of the technologies were used for inert gas purification (Kim et al., 2022). In addition, disadvantages like high reaction temperature, frequent regeneration, or low performance (or selectivity) of the oxygen-removal reaction restricted the application of these technologies to the removal of oxygen in the tail gas of the HPPO process. In the HPPO tail gas oxygen-removal reaction, the reaction between oxygen and hydrogen should be promoted, while propylene hydrogenation should be inhibited. Among several catalysts, the Pd catalyst usually showed good performance in hydrogen oxidation and selective hydrogenation. Although Pd is a good metal for catalytic reaction, the selectivity is not perfect. To improve its selectivity, people use many methods. The adsorption and desorption abilities to the olefine of Pd are important; Pd-containing bimetallic catalysts were usually used to solve the problem (Ravanchi et al., 2018; Shi et al., 2022). Pd/Al_2_O_3_-Mg and Pd/CaCO_3_ catalysts were reported having good selectivity in the olefine purification reaction because of the weaker Lewis acid centers on the surface (Cordoba et al., 2019). It indicates that alkali earth metals can weaken the acid centers in Pd catalysts, which helps to improve the selectivity of the catalysts. In hydrogen catalytic purification area, Pd has been widely used too. Pd has a very strong affinity for O_2_ and H_2_, the dissociative adsorption of H_2_ and O_2_ molecules on the surface of Pd catalysts occurs with little or no activation energy barrier (Adams and Chen, 2011). Pd-substituted Co_3_O_4_-ZrO_2_ was used for catalytic hydrogen combustion, and Pd improved H_2_ and O_2_ activation, which reduced the temperature of the reaction; the activation energy of the Pd catalyst was decreased by more than 2-fold (Singh et al., 2017). If the advantage of the Pd catalyst on hydrogen oxidation and selective hydrogenation can be combined, it should be a good choice for oxygen removal from unsaturated hydrocarbon (Kim et al., 2022).

In this work, a Pd catalyst was prepared, and it was used to remove oxygen (>2 vol %) in a propylene (>90 vol %) flow with hydrogen’s existence; the oxygen removal efficiency (ORE) and propane content in the product were investigated. Furthermore, alkali metals (Li, Na, and K) were used to modify the Pd catalyst; several characterizations were carried out to study the influence of the alkali metals on Pd species in the catalysts. It was found that oxygen could be removed from the propylene flow by Pd catalysts, alkali metals could enhance the performance of the Pd catalyst, and the effect followed the order of K > Na > Li. The characterization results explained the mechanism of the influence of alkali metals to the Pd catalyst.

## Experimental section

### Catalyst preparation

Pd/Al_2_O_3_ and alkali metal-modified Pd/Al_2_O_3_ catalysts were prepared by wetness impregnation. Aqueous solutions of PdCl_2_ (Shanghai Aladdin Biochemical Technology Co., Ltd.) and alkali metal nitrate of Li, Na, or K (Sinopharm Chemical Reagent Beijing Co., Ltd.) were added to Al_2_O_3_ (Sinopec Catalyst Co., Ltd.) support. After impregnation, the samples were dried at 100°C for 10 h and then calcined at 500°C for 4 h. The obtained catalysts were denoted as Pd/A, Pd-Li/A, Pd-Na/A, and Pd-K/A. The content of Pd was 0.3 wt %, and alkali metals were added with the same alkali metal/Pd mole ratio.

### Catalyst characterization

Powder X-ray diffraction (XRD) was carried out on a Rigaku Ultima IV (Cu-Kα, λ = 0.154 nm, 3 KW). The textual characterization was obtained by N_2_-physisorption on a Micromeritics ASAP 2020 at 77 K; the data were processed by using the Brunauer–Emmett–Teller (BET) method. Hydrogen temperature-programmed reduction (H_2_-TPR) was carried out using a Micromeritics Autochem 2950 with 10 % H_2_/Ar. X-ray photoelectron spectroscopy (XPS) was carried out on a VG ESCALAB MK II spectrometer with an Al-Kα (1,486.6 eV) photon source. The morphology of the catalysts was carried out on a JEOL JEM-2100UHR transmission electron microscopy (TEM).

### Catalyst test

Hydrocarbon oxygen removal tests were carried out on a fixed-bed reaction system. 1 ml catalyst was filled in a stainless steel reactor (I.D. = 6 mm). Before the test, the catalysts were pretreated in a H_2_ flow (15 sccm) at 150°C for 2 h. The tests were performed at 0.5 MPa and a LHSV 4,000 h^−1^. A gas mixture was injected into the reactor. The reaction products were separated in a separator, then the gas was analyzed using gas chromatography (GC, Agilent 7890B) with a flame ionization detector (FID) and two thermal conductivity detectors (TCD). The oxygen-removal performance was presented by oxygen removal efficiency (ORE), which was defined by the following formula:

$$ORE=\left(1-C_{O2\mathrm{-P}}/C_{O2\mathrm{-F}}\right){}^{*}100\%$$

$$C_{O2\mathrm{-P}}\mathrm{=content}\;\mathrm{of}\;\mathrm{oxygen}\;\mathrm{in}\;\mathrm{the}\;\mathrm{product}$$

$$C_{O2\mathrm{-F}}\mathrm{=content}\;\mathrm{of}\;\mathrm{oxygen}\;\mathrm{in}\;\mathrm{the}\;\mathrm{feed}$$

## Results and discussions

### Oxygen-removal performance

**FIGURE 1:**
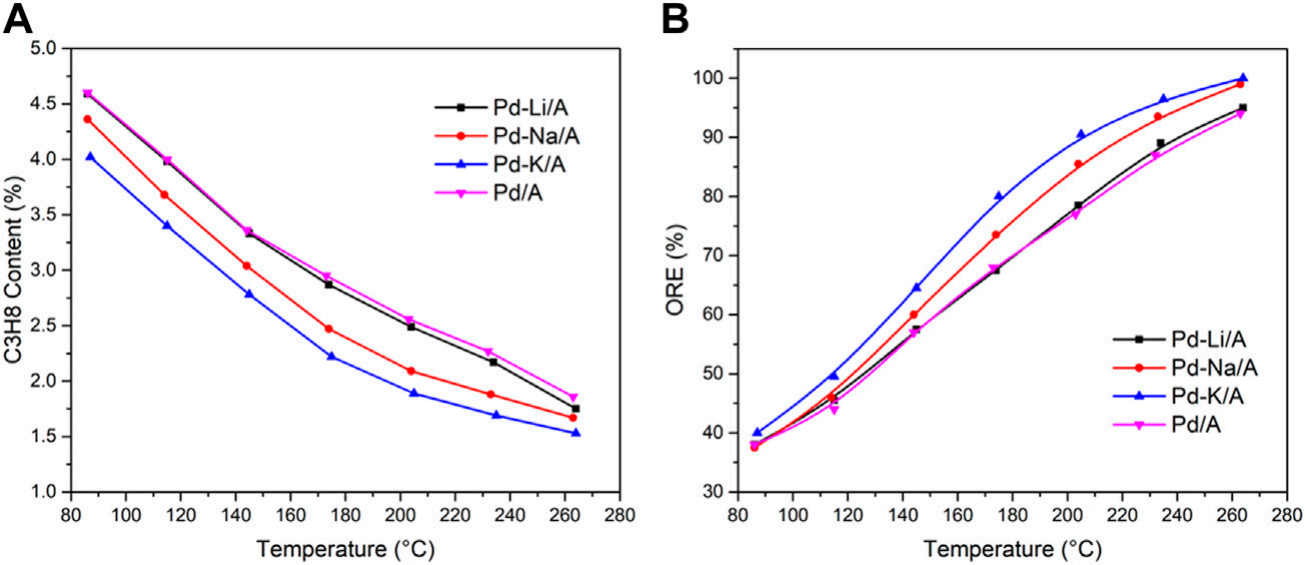
Oxygen removal efficiency **(A)** and content of propane in the products **(B)** for different catalysts.

In order to investigate the oxygen-removal performance of Pd/A and Pd-M/A (M = Li, Na or K) with the existence of hydrogen, a gas mixture of propylene (94 vol %), oxygen (2 vol %), and hydrogen (4 vol %) was introduced into the reactor after the catalysts were reduced. Figure 1A shows the oxygen removal efficiency (ORE) of different catalysts. The Pd/A catalyst had an ORE lower than 40 % at 80°C and achieved 94 % at 265°C. After the catalyst was modified by alkali metals, the performance of the Pd catalyst was enhanced remarkably. The Pd-Li/A had a similar performance with Pa/A between 80 and 265°C. Pd-Na/A and Pd-K/A performed better than Pd/A at the same temperature, and they achieved a 100% conversion at 265°C. Hydrogen also reacted with propylene while it reacted with oxygen, the byproduct was propane. Figure 1B shows propane content in the product gas. All catalysts had a similar trend between 80 and 265°C. Pd/A and Pd-Li/A had the same performance, while Pd-Na/A and Pd-K/A produced less propane in the reaction at the same temperature. According to the reaction results, the modification by alkali metals enhanced the performance of the Pd catalyst for the reaction of hydrogen and oxygen, while the reaction of hydrogen and propylene was restrained. The promotion effects seemed dependent on the chemical nature of the alkali metals, the order was K > Na > Li.

### Structural feature studies

**FIGURE 2:**
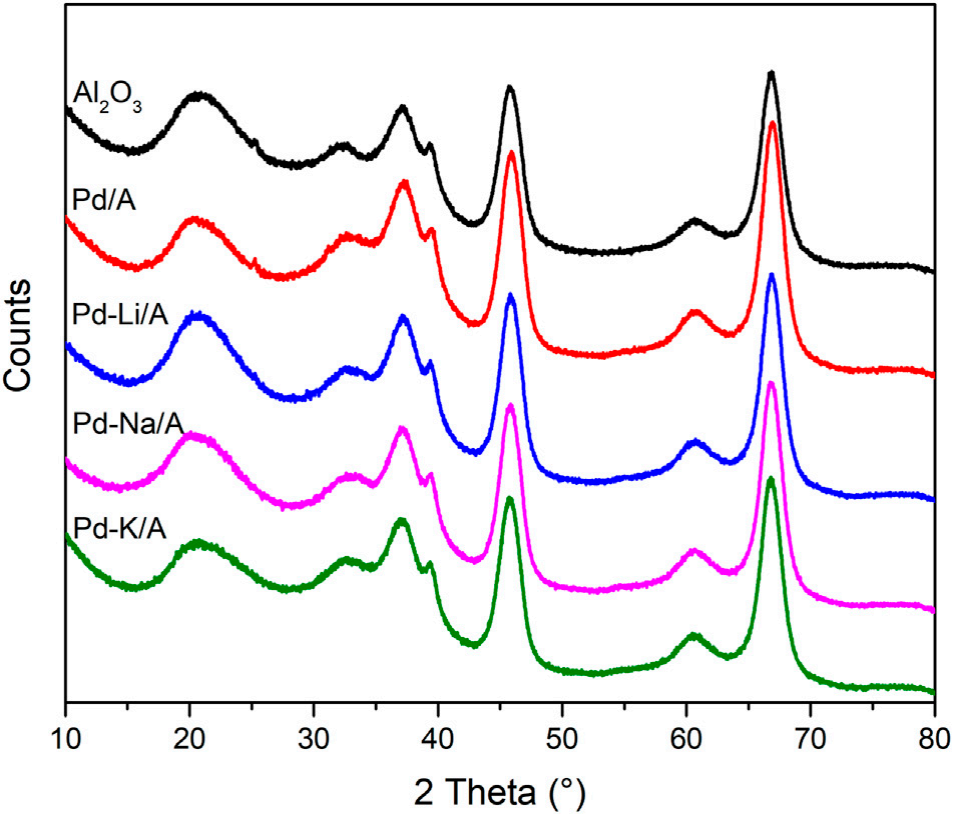
XRD patterns of Al_2_O_3_, Pd/A, and alkali metal (Li, Na, and K)-modified catalysts.

**FIGURE 3:**
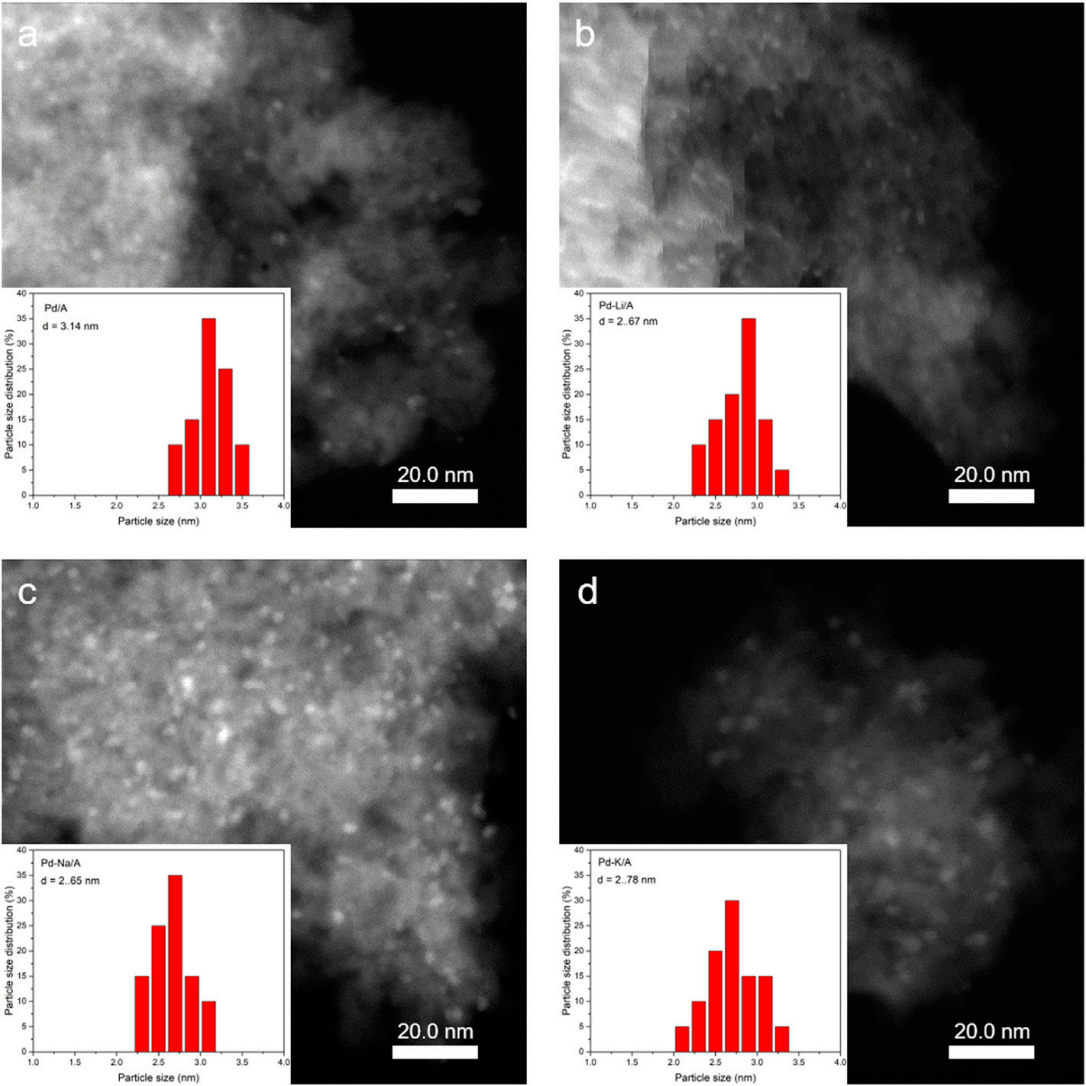
TEM images of **(A)** Pd/A, **(B)** Pd-Li/A, **(C)** Pd-Na/A, and **(D)** Pd-K/A catalysts.

The XRD patterns of Al_2_O_3_, Pd/A, and alkali metal-modified Pd catalysts are shown in Figure 2. The pattern of Al_2_O_3_ indicated that it was γ-Al_2_O_3_. In the patterns of Pd/A and alkali metal-modified Pd catalysts, there was no observable peak that belonged to Pd or other species, which indicated that Pd and alkali metals were highly dispersed on the supports (Peralta-Robledo et al., 2020). The pore structure information of Al_2_O_3_ support, Pd/A, and alkali metal-modified Pd catalysts are listed in Table 1. Compared with Al_2_O_3_ support, the surface area of Pd/A and Pd-M/A (M = Li, Na, K) decreased a little bit, which might be ascribed to the metal species covering the surface of the support. Figure 3 shows the TEM image of Pd/A and alkali metal-modified Pd catalysts. As shown in the images, Pd species were dispersed on the surface of the catalysts, no big block was observed. The particle size of Pd species on the surface of catalysts was statistically analyzed. The results showed that the particle size distribution of the catalysts was between 2.0 and 3.5 nm. The average particle size of Pd/A was 3.14 nm, while it was 2.67, 2.56, and 2.78 nm, respectively, over the Pd-Li/A, Pd-Na/A, and Pd-K/A. It indicated that the alkali metal modification helped the Pd species to form smaller particles, which improved the distribution of the Pd species on the surface of the catalysts.

**TABLE 1:** Pore structure information of Al_2_O_3_ support, Pd/A, and alkali metal-modified Pd catalysts.

Sample	Surface area (m^2^·g^−1^)	Pore size (nm)	Pore volume (cm^3^·g^−1^)
Al_2_O_3_	145.1	10.1	0.65
Pd/A	139.5	10.0	0.65
Pd-Li/A	140.6	10.0	0.63
Pd-Na/A	136.7	9.9	0.64
Pd-K/A	135.5	10.0	0.66

### H_2_-TPR studies

**FIGURE 4:**
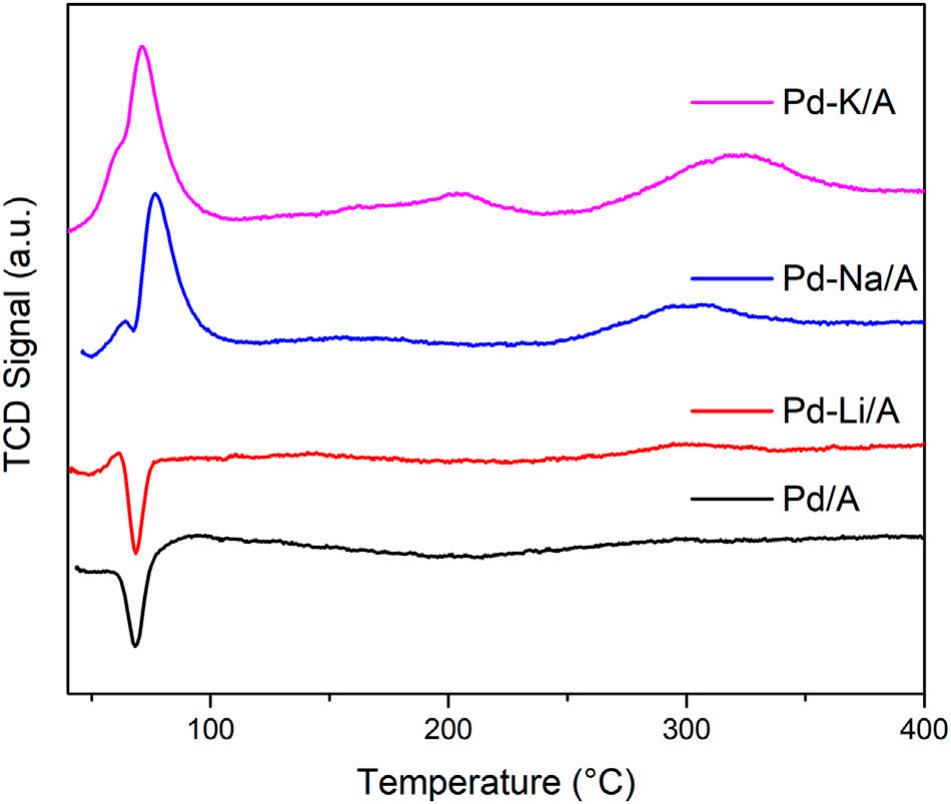
H_2_-TPR profiles of Pd/A and alkali metal (Li, Na, and K)-modified catalysts.

To study the reducibility of the Pd/A and alkali metal (Li, Na, and K)-modified catalysts, the hydrogen temperature-programmed reduction was carried out. Figure 4 shows the H_2_-TPR profiles of the catalysts, the differences of the profiles of the catalysts are obvious. Pd/A and Pd-Li/A presented negative peaks at 68°C, and Li-modified Pd catalysts presented small positive peaks at around 63°C; otherwise, Pd-Na/A and Pd-K/A presented big positive peaks at 76 and 72°C, respectively. The positive peaks of the profiles could be ascribed to the hydrogen consumption of the reduction of Pd oxide, while the negative peaks are ascribed to the hydrogen generation of the decomposition of the β-PdH formed during the reduction at lower temperature (Batista et al., 2001). Pd oxide is easily reduced by H_2_ at room temperature; Pd/A showed only a negative peak, which ascribed to the β-PdH decomposition. Positive peaks appeared in the profiles of alkali-modified Pd catalysts, although there was still a sharp negative peak in the profile of Pd-Li/A. The positive peak could be ascribed to the interaction between Pd and Li, but the interaction was too weak to affect the Pd species much. The positive peaks in the profiles of Pd-Na/A and Pd-K/A were obvious, but the negative peak disappeared. In addition to the small positive peak at 63°C, a big peak appeared at a higher temperature, which responded to the reduction of Pd oxide. These phenomena indicated that there was an interaction between the Pd species and alkali metals, which affected the reducibility of Pd oxide and the formation of β-PdH on the surface of Pd species. The intensity of the interaction followed the order of K > Na > Li. According to the rule, Pd-K/A had the least β-PdH which responded to hydrogenation of propylene, which could well coincide with the reaction results.

### XPS studies

**FIGURE 5:**
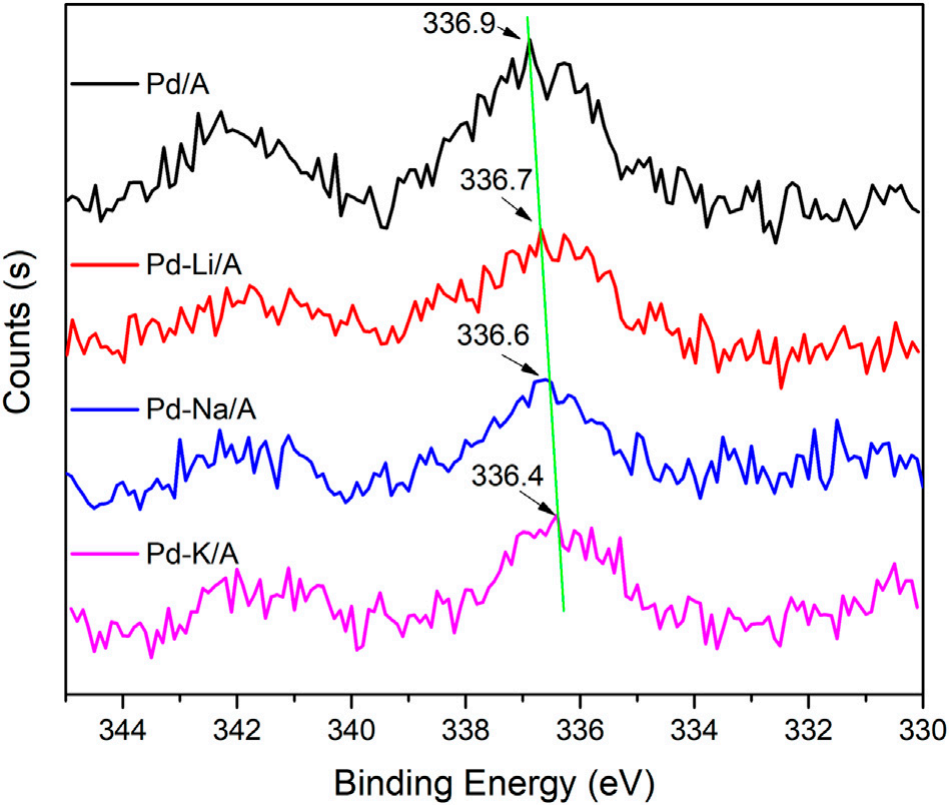
Pd 3d XPS spectra of Pd/A and alkali metal (Li, Na, and K)-modified catalysts.

XPS was carried out to investigate the electron structure information of Pd species on the surface of the catalysts and the effect of alkali modification. The spectra of Pd 3d for Pd/A and alkali metal (Li, Na, and K)-modified Pd catalysts are displayed in Figure 5. In the curve of Pd/A, the peak at 336.9 eV could be ascribed to PdO, which was due to the oxidation in the production process (Batista et al., 2001; Li et al., 2016). With the addition of alkali metals, the peak of PdO shifted to a lower binding energy. The top of the peaks appeared at 336.7, 336.6, and 336.4 eV over Pd-Li/A, Pd-Na/A, and Pd-K/A, respectively. The results indicated that alkali metals played the role of the electron donor, who had interaction with the Pd species in the modified catalysts. The decrease of the binding energy indicated that the addition of alkali metals increased the electron density of Pd species, and the electron donation effect became stronger with the chemical nature of the alkali metals, following the order of K > Na > Li (Liotta et al., 1996a; Li et al., 2016). The binding energy of Pd-Li/A decreased the least among the modified catalysts, it could be attributed such that Li gives out the least electron for the strongest electronegativity among the alkali metals, and the interaction between Pd and Li could be weaker than other alkali metals. Pd species were the main active sites of the reaction between hydrogen and oxygen or hydrogen and propylene; the negatively charged Pd species should perform differently. Adsorption of reactant molecules on the active sites is an important step during the reaction, and the electron density of Pd species could affect the adsorption to change the reaction performance (Liotta et al., 1996b). Adsorption of propylene on the active site by π bond should be decreased for the electron density increase of the negatively charged Pd species. On the other hand, the negatively charged Pd species could enhance the adsorption of oxygen because of the electron transfer from Pd species to the anti-bonding π* orbital of oxygen molecules (Huang and Leung, 2011; Matveev et al., 2015; Xu et al., 2019). This change may promote the reaction between hydrogen and oxygen, while the reaction between hydrogen and propylene would be decreased. For the difference of interaction between Pd species and alkali metals, the activity of the modified Pd-based catalysts should follow the order of Pd-K/A > Pd-Na/A > Pd-Li/A, the results were consistent with the performance of the oxygen removal test.

### Stability studies

The aforementioned comparative experiments and characterizations showed that Pd-K/A was the best item among the studied catalysts; then, a long-term experiment was carried out to investigate the stability of the Pd-K/A catalyst. The reaction was performed on a fixed-bed reactor in which 1 ml Pd-K/A catalyst was loaded. A gas mixture consisting of propylene (94 vol %), oxygen (2 vol %), and hydrogen (4 vol %) was used as model feed. After the reaction ran for 150 h, steam (about 2 mol %) was introduced into the reactor to investigate the influence of water to the oxygen removal reaction. The test was performed for 200 h at the conditions of T = 240°C, *p* = 0.5 MPa, and a LHSV = 4,000 h^−1^. When the temperature of the catalyst stabilized at 240°C, the gas product was analyzed by using gas chromatography every 2 h.

**FIGURE 6:**
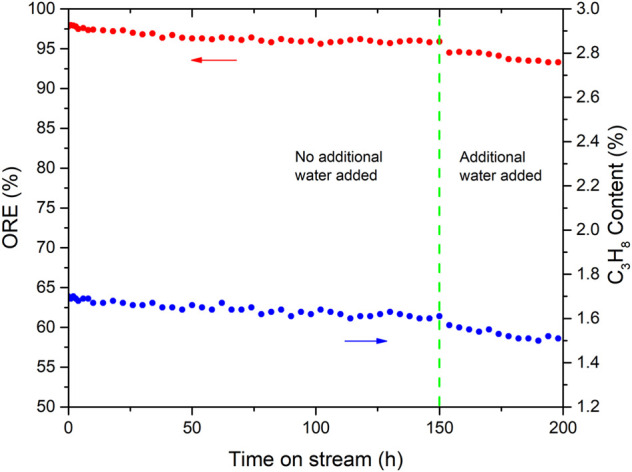
Long-term performance of the Pd-K/A catalyst (T = 240°C, p = 0.5MPa, LHSV = 4,000 h^−1^).

Figure 6 shows the ORE and the C_3_H_8_ content in the gas product during the long-term experiment. In the initial stage, the ORE of the reaction was above 97% at the first 24 h, and the content of C_3_H_8_ in the product was about 1.68%. The reason for the perfect performance was that the fresh Pd-K/A catalyst had a relatively high activity at the beginning, and the reaction of H_2_ with O_2_ and H_2_ with propylene were all promoted. After the initial stage, the activity of the Pd-K/A catalyst became stable; the ORE remained at 95–97%, while the content of C_3_H_8_ in the product remained at 1.55–1.65%. When the steam was introduced into the reaction at 150 h, the ORE showed a downward trend, the content of C_3_H_8_ in the product went down too, which indicated that the activity of the Pd-K/A catalyst was affected. In the oxygen-removal process, the product of the reaction between H_2_ and O_2_ is H_2_O. It means that there was always water in the reaction system. In Figure 6, the ORE of the reaction before 150 h remained above 95 % stably, but the ORE began going down while the additional water (2 % steam) was introduced into the reactor. It indicated that water could influence the activity of the Pd-K/A catalyst; water molecules might adsorb on the surface of the catalyst to cover the active site, leading to the activity falling. According the result of the stability experiment, the quantity of the water involving the reaction was important. Although water was continuously generated during the reaction, a small amount of water could not affect the activity of the Pd-based catalyst. When the additional water was introduced in the system, the amount of water molecules around the active sites increased, which resulted in the formation of a water film that covered the active site (Kim et al., 2022).

## Conclusion

Pd and alkali metal-modified Pd catalysts could remove oxygen in the propylene flow with hydrogen existence by catalytic reaction between oxygen and hydrogen, and the reaction between propylene and hydrogen could be inhibited to a certain extent. The addition of alkali metals could facilitate the reducibility of Pd species and inhibit the formation of β-PdH which was responsible for propylene hydrogenation. The electron density of the Pd atom was increased by the metal–metal interaction to form negatively charged Pd species which adsorbed oxygen molecules easier than propylene molecules. The improvement of alkali metals depended on the chemical nature of the alkali metals, and the order was K > Na > Li. The characterization results of Pd-K/A indicated that the strongest interaction between Pd and K contributed to its highest ORE and selectivity. The stability test showed that the Pd-K/A catalyst could perform well for a long time; the ORE could remain at 95–97% at 240°C. Water could influence the activity of the catalyst, but the quantity of water was important. Water formed during the reaction could not affect the catalyst, but additional water might weaken the activity of the catalyst for the water molecules adsorbed on the surface covering the active site of the catalyst.

## Data Availability

The original contributions presented in the study are included in the article/Supplementary Material; further inquiries can be directed to the corresponding authors.
